# Decoupling Transparency and Electron Injection in Ultrathin Ag Electrodes Using an Al/MoO_3_ Double Seed Layer for Transparent Quantum Dot Light‐Emitting Diodes

**DOI:** 10.1002/nap2.70283

**Published:** 2026-09-03

**Authors:** Yeyun Bae, Kyoungeun Lee, Kyunghoon Lee, Jaeyeop Lee, Jiyoon Oh, Jiwoong Yang, Chansu Kim, Jeongkyun Roh

**Affiliations:** ^1^ Department of Electrical Engineering Pusan National University Busan Republic of Korea; ^2^ Department of Energy Science & Engineering DGIST Daegu Republic of Korea; ^3^ Extreme Process Control Group Korea Institute of Industrial Technology (KITECH) Busan Republic of Korea; ^4^ Energy Science and Engineering Research Center Daegu Gyeongbuk Institute of Science and Technology (DGIST) Daegu Republic of Korea; ^5^ DGIST InnoCORE Energy Materials Commercialization (E‐MatAX) Research Center Daegu Gyeongbuk Institute of Science and Technology (DGIST) Daegu Republic of Korea; ^6^ MOPLAT Seoul Republic of Korea

**Keywords:** charge injection efficiency, large‐area fabrication, quantum dot light‐emitting diodes, quantum dots, seed layer, transparent electrode

## Abstract

There is growing interest in transparent quantum dot light‐emitting diodes (Tr‐QLEDs) for window‐type displays. For such devices, the top transparent electrode must simultaneously provide high optical transmittance, low sheet resistance, and efficient charge injection. Ultrathin Ag films are promising because of their high conductivity and transparency, but they tend to grow via the Volmer–Weber mode, resulting in poor continuity and increased resistance. Although seed layers have been widely used to improve Ag film continuity, their influence on charge injection in practical devices has received limited attention. Here, an Al/MoO_3_ double seed layer is introduced, in which MoO_3_ provides high transmittance and low sheet resistance, while Al enhances electron injection. This strategy enables continuous 10 nm Ag films with a transmittance of 63.48% and a sheet resistance of 7.35 Ω sq^−1^. Tr‐QLEDs incorporating the Al/MoO_3_ double seed layer exhibit a twofold increase in external quantum efficiency and a threefold improvement in operational lifetime compared with devices employing a MoO_3_ single seed layer. Furthermore, large‐area Tr‐QLEDs are demonstrated on a 2 × 2 inch substrate, highlighting the potential of this electrode strategy for scalable transparent displays.

## Graphical abstract

Transparent quantum dot light‐emitting diodes (Tr‐QLEDs) employing an Al/MoO_3_ double seed layer decouple optical transparency from electron injection in ultrathin Ag electrodes. The functionally separated seed layers enable low optical loss and efficient charge injection simultaneously, resulting in improved electroluminescent performance, enhanced operational stability, and uniform large‐area devices suitable for scalable transparent display technologies.

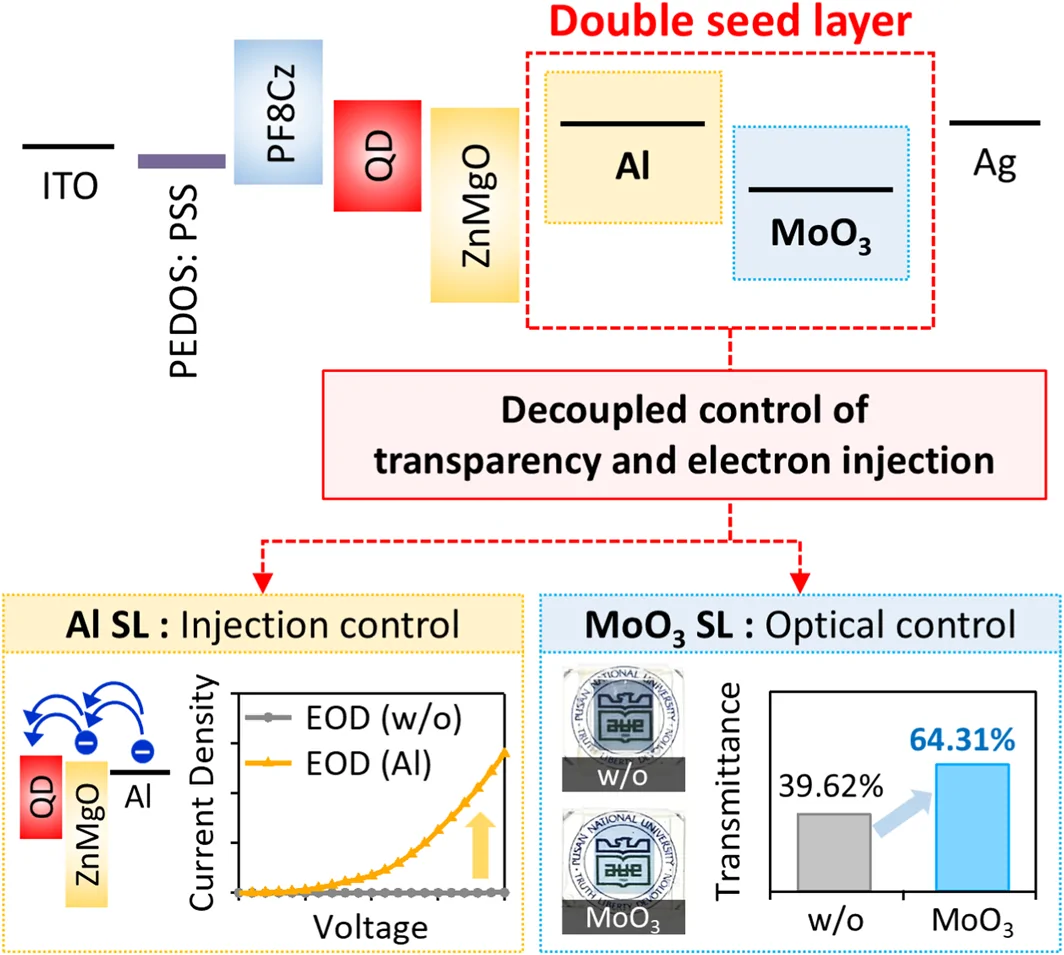

## 1 Introduction

Quantum dot light‐emitting diodes (QD‐LEDs) have attracted significant attention as next‐generation display technologies owing to their unique advantages, including a narrow full‐width at half‐maximum (FWHM) [1, 2], high color purity, solution processability [3], and tunable emission wavelengths enabled by precise size control [4, 5]. Over the past decade, QD‐LEDs have been extensively investigated and have progressed toward commercialization with respect to efficiency, operational stability, and patterning technologies [6, 7, 8, 9, 10, 11, 12, 13, 14, 15].

Building on these advances, recent research has increasingly focused on transparent QD‐LEDs (Tr‐QLEDs), which offer new opportunities for next‐generation optoelectronic devices, particularly in advanced lighting and window‐type display applications. In such devices, the transparent top electrode plays a crucial role in determining both optical transparency and electrical performance. To date, various transparent electrode materials, including transparent conductive oxides (TCOs) [16, 17, 18], metal nanowires [19, 20, 21], carbon nanotubes [22, 23], conductive polymers [24, 25], graphene [26, 27], and ultrathin metal films [28, 29] have been explored. However, each material class has inherent drawbacks. TCOs (e.g., indium tin oxide (ITO), indium zinc oxide (IZO)) are typically deposited by sputtering, which often requires high‐temperature processing, can damage underlying layers, and increases production costs. Solution‐processed electrodes such as metal nanowires, carbon nanotubes, and conductive polymers offer low‐temperature processing and printability; however, the use of solvent‐based inks and wet deposition can chemically or physically damage underlying functional layers through dissolution, swelling, or interfacial contamination, thereby degrading device reproducibility and reliability. Moreover, these electrodes often suffer from high surface roughness and weak interfacial adhesion, which can degrade device reliability. In particular, conductive polymers such as PEDOT:PSS exhibit limited conductivity and poor long‐term stability because of their hygroscopic and acidic nature, which can lead to corrosion of adjacent layers and gradual conductivity degradation. Graphene electrodes generally rely on transfer and lamination processes, making it difficult to achieve uniform large‐area films with low defect density; furthermore, their electrical performance often depends on chemical doping that can be unstable over time, further limiting practical implementation.

By contrast, thermally evaporated ultrathin metal films can provide high conductivity while maintaining good optical transparency. In addition, vacuum deposition enables uniform and scalable large‐area film formation, making ultrathin metal electrodes particularly attractive for the commercialization of Tr‐QLEDs. Among candidate metals, silver (Ag) is particularly promising because of its excellent conductivity and high transparency in the visible range. Nevertheless, ultrathin Ag films inherently suffer from a trade‐off between optical transmittance and electrical continuity. Due to the Volmer–Weber growth mode [30, 31], Ag tends to form discontinuous island‐like morphologies at low thicknesses. While such discontinuity can increase optical transparency, it simultaneously results in high sheet resistance and poor electrical conduction. Therefore, achieving continuous and conductive ultrathin Ag films at minimal thickness remains a critical challenge for high‐performance Tr‐QLEDs.

To address this issue, previous studies have introduced seed layers to promote continuous Ag film formation. Seed layers such as molybdenum trioxide (MoO_3_) [32, 33], gold (Au) [34], and aluminum (Al) [35, 36] have been employed to facilitate Ag nucleation and coalescence. These approaches can improve optical transmittance and/or electrical conductivity by lowering the percolation threshold and enhancing film continuity. However, most prior studies have primarily evaluated electrode performance in terms of transmittance and sheet resistance, with relatively limited attention paid to charge injection characteristics in complete device structures. In practical Tr‐QLED operation, the transparent electrode must not only provide high transparency and low resistance but also enable efficient carrier injection—particularly electron injection when used as a cathode—to maximize luminance, efficiency, and operational stability.

In this study, we propose an Al/MoO_3_ double seed layer architecture for ultrathin Ag transparent electrodes, in which MoO_3_ provides an opto‐electrically favorable growth template while Al primarily enhances electron injection when the transparent electrode functions as a cathode. Specifically, the MoO_3_ interlayer enables the formation of high‐quality ultrathin Ag films with both high optical transmittance and low sheet resistance, while introducing an additional ultrathin Al layer at the interface improves electron injection without sacrificing the optical and electrical advantages afforded by MoO_3_. As a result, the double‐seed design enables continuous Ag films even at a thickness of 10 nm, simultaneously delivering low sheet resistance, high transmittance, and enhanced electron injection. Tr‐QLEDs fabricated with the double seed layer exhibit a twofold increase in luminance and external quantum efficiency (EQE) compared with devices employing a MoO_3_ single seed layer, along with a threefold improvement in operational lifetime. Furthermore, large‐area Tr‐QLEDs (2 inch × 2 inch) are demonstrated, highlighting the scalability and commercialization potential of the proposed electrode strategy. Overall, this work demonstrates that the proposed double‐seed strategy effectively decouples the intrinsic trade‐off among optical transparency, electrical conductivity, and electron injection in ultrathin Ag electrodes, providing a practical route toward scalable, high‐performance Tr‐QLEDs.

## 2 Results and Discussion

Figure 1a shows the schematic structure of the Tr‐QLEDs fabricated in this study. The devices consist of multiple functional layers, including a hole injection/transport layer (HIL/HTL) composed of PEDOT: PSS and PF8Cz, an emissive layer (EML) composed of quantum dots (QDs), an electron transport layer (ETL) composed of ZnMgO nanoparticles (NPs), and transparent electrodes. The bottom transparent electrode is indium tin oxide (ITO), which is widely used due to its high optical transmittance and low sheet resistance. For the top transparent electrode, thermally evaporated ultrathin metal films are preferred for practical applications because they can be deposited as uniform, large‐area films through a simple and scalable process. Among them, Ag thin films are commonly employed due to their outstanding conductivity and transparency. However, ultrathin Ag films often suffer from poor film continuity because their early‐stage growth typically proceeds via island nucleation. To understand this behavior, the growth mechanism of metal thin films must be considered.

**FIGURE 1:**
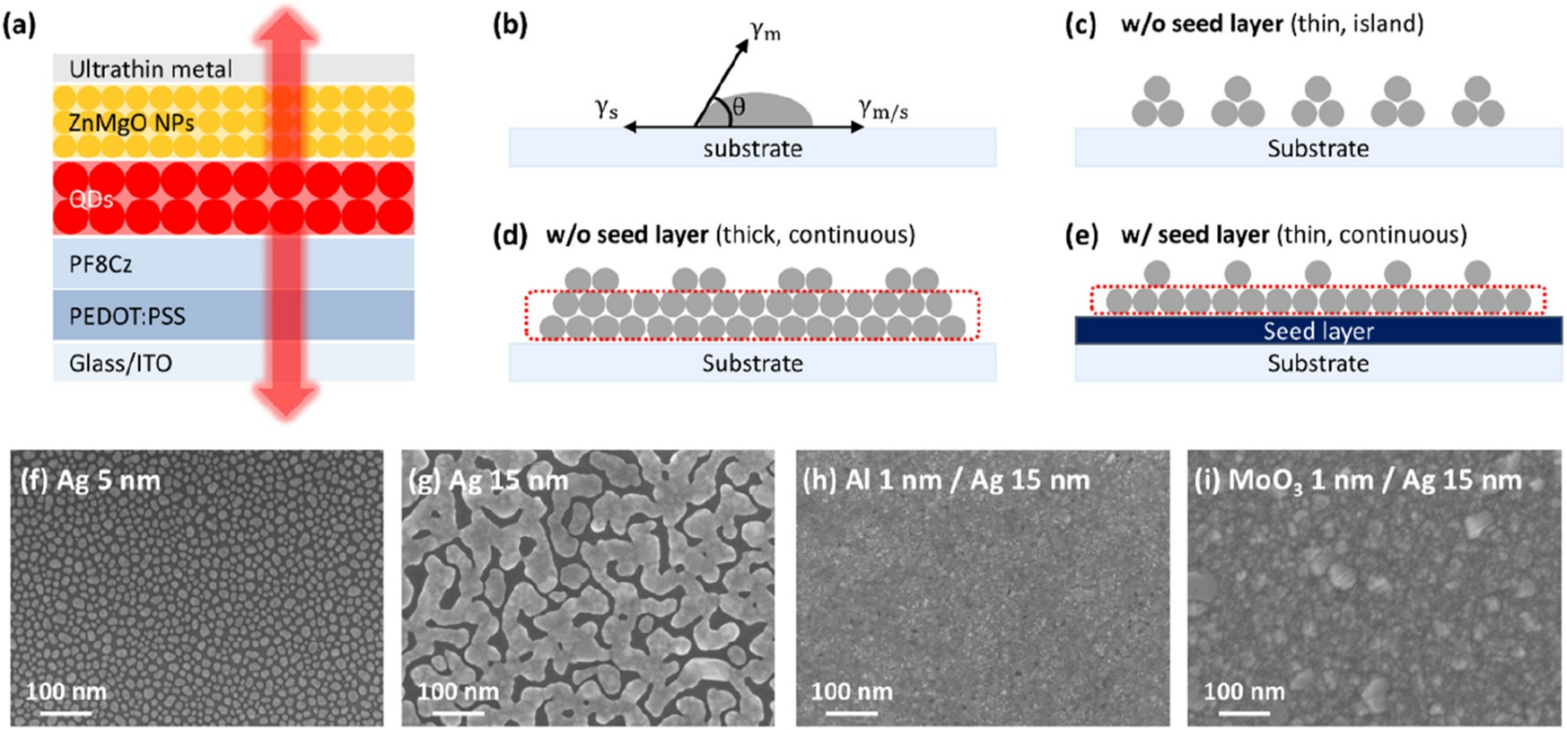
(a) Schematic device structure of the Tr‐QLED. (b) Schematic illustration of Volmer–Weber growth. Evolution of metal thin‐film growth without a seed layer: (c) discontinuous island formation at low thickness and (d) coalescence into a continuous film at higher thickness. (e) Effect of a seed layer in promoting earlier coalescence and enabling a continuous film at reduced thickness. (f, g) SEM images of Ag films with increasing thickness: (f) 5 nm and (g) 15 nm. (h, i) SEM images of Ag films deposited on different seed layers: (h) Al and (i) MoO_3_.

The growth mechanism of metal thin films is governed by the energetic balance among the substrate surface energy ($\gamma_{s}$), the metal surface energy ($\gamma_{m}$), and the metal/substrate interfacial energy ($\gamma_{m/s}$) [37, 38], as illustrated in Figure 1b. The adhesion energy (*E*_adh_) [39] between the deposited metal and the substrate can be expressed as:

(1) $${E_{\text{adh}}=\gamma }_{m}+\gamma_{s}-\gamma_{m/s}.$$

When the adhesion energy is smaller than the cohesive interaction between metal atoms (i.e., ${E_{\text{adh}}<2\gamma }_{m}$), metal‐metal bonding is favored over metal‐substrate bonding, leading to three‐dimensional island nucleation via the Volmer‐Weber growth mode. Rearranging Equation (1) yields:

(2) $$\gamma_{s}<\gamma_{m}+\gamma_{m/s},$$

which indicates that island growth occurs when the effective substrate surface energy is lower than the sum of the metal surface energy and the interfacial energy.

For Ag films, the cohesive attraction between adjacent Ag atoms ($\gamma_{m}\;\approx$ 1.25 J m^−2^) [40] is generally much stronger than the adhesion between Ag and typical underlying dielectric/oxide substrates (e.g., ZnO, glass, and ITO), whose surface energies are significantly lower (< 0.1 J m^−2^) [40, 41, 42]. Consequently, the condition described in Equation (2) is readily satisfied, and the initial Ag growth follows the Volmer–Weber mode, resulting in the formation of isolated islands (Figure 1c). With continued deposition, these islands gradually coalesce and merge, eventually forming a continuous film (Figure 1d). This coalescence process explains why Ag typically requires a relatively high critical thickness to achieve electrical continuity.

To overcome this limitation, seed layers are commonly introduced to promote continuous Ag film formation by increasing the metal–substrate adhesion energy. In practice, a seed layer can reduce the interfacial energy ($\gamma_{m/s}$) and/or effectively increase the substrate surface energy ($\gamma_{s}$), thereby relaxing the island‐growth condition and enabling continuous Ag films at a reduced thickness. Figure 1e schematically illustrates how a seed layer facilitates continuous thin‐film growth at thinner Ag thicknesses.

The surface morphology of Ag films with different thicknesses was examined using scanning electron microscopy (SEM). As shown in Figure 1f, a 5 nm‐thick Ag film exhibits discontinuous island‐like features, consistent with Volmer–Weber growth. In contrast, at a larger thickness (Figure 1g), the Ag film becomes much more continuous, indicating improved electrical connectivity.

Furthermore, the effects of different seed layers on Ag film morphology were investigated. Figure 1h,i show SEM images of Ag films deposited on Al and MoO_3_ seed layers, respectively. Compared with Ag films deposited directly on the substrate without a seed layer, the introduction of either seed layer improves film uniformity and promotes more continuous Ag coverage at reduced thickness. These results highlight the important role of seed layers in controlling ultrathin metal film growth and improving film continuity.

Following the analysis of Ag thin‐film morphology, the optical and electrical properties of the Ag films with and without a seed layer were evaluated as a function of Ag thickness. Figure 2a summarizes the optical transmittance and sheet resistance of Ag films with different thicknesses, comparing films deposited directly on the substrate and those grown on Al or MoO_3_ seed layers. The corresponding transmittance–wavelength spectra for each thickness and seed layer are provided in Supporting Information S1: Figure S1, and the transmittance at 623 nm together with the sheet resistance values are summarized in Table 1. To ensure measurement reliability, each sheet resistance value represents the average of three independent measurements. As the Ag film thickness decreases, the optical transmittance increases while the sheet resistance increases, reflecting the inherent trade‐off between transparency and conductivity in ultrathin metal electrodes. Without a seed layer, measurable sheet resistance was obtained only for the 15 nm Ag films, whereas thinner films (< 10 nm) were discontinuous and could not be electrically characterized (Supporting Information S1: Figure S2).

**FIGURE 2:**
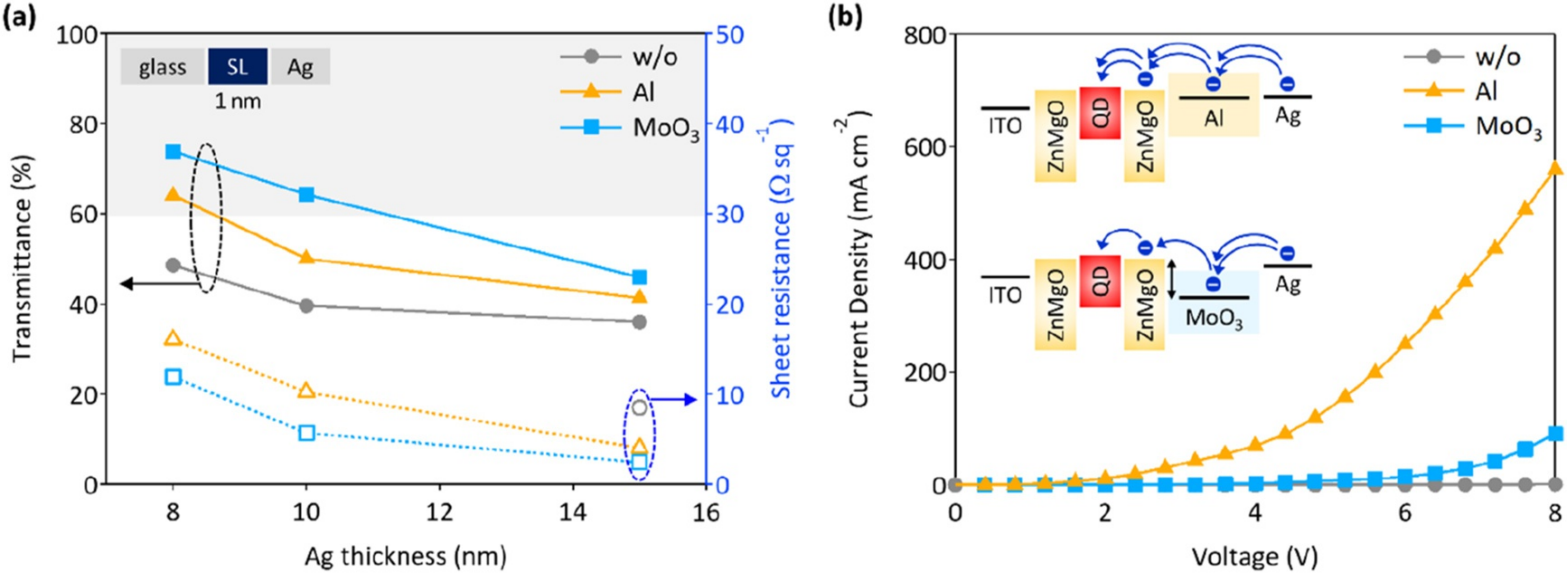
(a) Optical transmittance and sheet resistance of Ag films as a function of thickness, comparing films deposited without a seed layer (SL) and those deposited on Al or MoO_3_ seed layers. (b) Current density–voltage (*J–V*) characteristics of electron‐only devices (EODs) incorporating 15 nm Ag electrodes deposited without a seed layer or on Al or MoO_3_ seed layers. The inset shows the corresponding energy band diagrams of the EODs for each seed layer.

**TABLE 1:** Transmittance and sheet resistance of Ag films as a function of thickness (sheet resistance values represent the average of three independent measurements).

Seed layer	Ag thickness (nm)	Transmittance (%)	Sheet resistance (Ω sq^−1^)
w/o	8	48.64	—
	10	39.62	—
	15	36.01	8.52
Al	8	64.16	16.08
	10	50.07	10.23
	15	41.35	4.03
MoO_3_	8	73.91	11.92
	10	64.31	5.68
	15	45.96	2.44

In contrast, introducing either Al or MoO_3_ seed layer enabled earlier film percolation, allowing continuous Ag formation and measurable sheet resistance even at an Ag thickness of 8 nm. This reduced percolation threshold allows the use of a thinner Ag layer while maintaining electrical conductivity. Notably, Ag films deposited on the MoO_3_ seed layer exhibited the highest optical transmittance at the same thickness. For example, at an Ag thickness of 10 nm, the electrode with an Al seed layer showed a transmittance of 50.07%, whereas that with a MoO_3_ seed layer exhibited a higher transmittance of 64.31%. Interestingly, although the Ag film seeded by 1‐nm Al exhibits a more uniform morphology in SEM compared with the 1‐nm MoO_3_‐seeded case, the optical transmittance is higher for the MoO_3_‐seeded electrode. This suggests that the transmittance of ultrathin metal electrodes is not solely governed by morphological continuity, but is also strongly influenced by the optical loss introduced by the seed layer and the resulting interfacial optical boundary conditions. Specifically, metallic Al has a relatively large extinction coefficient (*k*) in the visible range, which can induce additional parasitic absorption and increase effective reflectance even at sub‐nanometer thickness, thereby lowering the overall transmittance despite improved morphology. In contrast, MoO_3_ is a wide‐bandgap oxide with a smaller extinction coefficient in the visible range than metallic Al, and thus is expected to impose a smaller optical penalty by minimizing parasitic absorption associated with the seed layer. Moreover, the MoO_3_ interlayer can modify the interfacial optical impedance and effective reflectance of the MoO_3_/Ag stack, which can further contribute to enhanced transmittance. As a result, the MoO_3_‐seeded electrode maintains higher optical transmittance despite the relatively less uniform morphology observed in SEM.

Beyond the optical transmittance and sheet resistance of the transparent electrodes, charge injection at the electrode/transport‐layer interface critically influences device performance. To evaluate electron injection characteristics, electron‐only devices (EODs) were fabricated using 15 nm Ag films deposited on different seed layers. As shown in Figure 2b, the device without a seed layer exhibited limited electron injection, which can be attributed to the relatively poor interfacial contact and increased series resistance associated with seed‐free Ag deposition. In comparison, the device with an Al seed layer demonstrated enhanced electron injection, consistent with favorable band alignment. To further examine the cathode electronic properties, ultraviolet photoelectron spectroscopy (UPS) measurements were performed on the completed ZnMgO/electrode stacks (Supporting Information S1: Figure S3). The effective work functions of the ZnMgO/Ag, ZnMgO/Al/Ag, and ZnMgO/MoO3/Ag stacks were determined to be 4.70, 4.43, and 4.58 eV, respectively. Compared with the ZnMgO/Ag stack, the cathode structures incorporating seed layers exhibited lower effective work functions, which may be attributed to seed‐layer‐induced changes in the nucleation and growth behavior of the ultrathin Ag film. The ZnMgO/Al/Ag stack exhibited the lowest effective work function among the investigated cathode structures, which may be associated with the lower intrinsic work function of Al relative to MoO_3_ and indicates a more favorable condition for electron injection. This trend is consistent with the markedly higher current density observed in the Al‐seeded EOD. In contrast, the MoO_3_‐seeded device exhibited substantially lower electron current, indicating less favorable electron injection than the Al‐seeded device.

While the Al seed layer results in a lower optical transmittance than the MoO_3_ seed layer, it provides more efficient electron injection. In contrast, the MoO_3_ seed layer offers superior transmittance (and favorable sheet resistance) but exhibits inferior electron injection performance in device structures. To simultaneously achieve high optical transmittance, low sheet resistance, and enhanced electron injection, we introduced an Al/MoO_3_ double seed layer architecture (Figure 3a).

**FIGURE 3:**
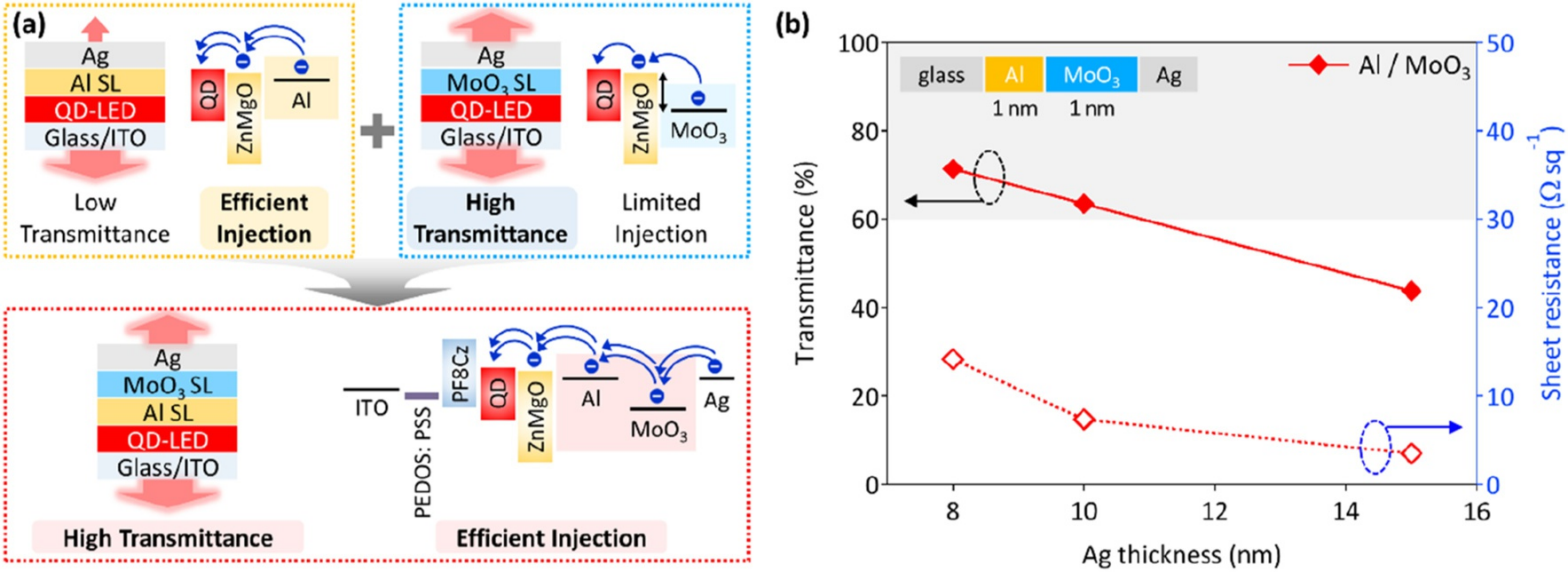
(a) Schematic illustration of the Al/MoO_3_ double seed layer strategy, highlighting the distinct roles of the Al and MoO_3_ interlayers in enhancing electron injection and preserving high optical transmittance, respectively. (b) Optical transmittance and sheet resistance of Ag films deposited on the Al/MoO_3_ double seed layer as a function of Ag thickness.

Figure 3b presents the transmittance and sheet resistance of Ag films deposited on the Al/MoO_3_ double seed layer as a function of Ag thickness, and the detailed values are summarized in Table 2. Similar to the single‐seed cases, the Al/MoO_3_ configuration enabled continuous film formation and measurable sheet resistance even at an Ag thickness of 8 nm. At 10 nm thickness, Ag films with the Al/MoO_3_ double seed layer exhibited a transmittance of 63.48% and a sheet resistance of 7.35 Ω sq^−1^. Compared with the MoO_3_ single seed layer, the double‐seed electrode shows a slightly lower transmittance and a higher sheet resistance; however, it outperforms the Al single seed layer in both properties. To verify the nominal thicknesses of the seed‐assisted ultrathin electrode stacks, atomic force microscopy (AFM) step‐height measurements were performed (Supporting Information S1: Figure S4). The measured step heights of the Al/Ag, MoO_3_/Ag, and Al/MoO_3_/Ag electrodes were approximately 11–12.5 nm, which agree well with the expected nominal thicknesses.

**TABLE 2:** Transmittance and sheet resistance of Ag films as a function of thickness (sheet resistance values represent the average of three independent measurements).

Seed layer	Ag thickness (nm)	Transmittance (%)	Sheet resistance (Ω sq^−1^)
Al/MoO_3_	8	71.41	14.17
	10	63.48	7.35
	15	43.78	3.51

Notably, the Al/MoO_3_ double‐seed electrode maintains a transmittance comparable to that of the MoO_3_ single seed, despite the presence of the additional Al layer. To further clarify this optical behavior, the reflectance spectra of the corresponding electrodes were measured, as shown in Supporting Information S1: Figure S5. The Al/MoO_3_/Ag electrode exhibited substantially lower reflectance than the Al/Ag electrode and maintained a reflectance comparable to that of the MoO_3_/Ag electrode. This result suggests that the MoO_3_ interlayer modifies the optical boundary conditions of the electrode stack and suppresses interfacial reflection, thereby mitigating the optical penalty associated with the Al layer. Similar reflection‐suppression effects of dielectric interlayers have been reported for ultrathin metal‐film‐based transparent electrodes [39, 43, 44, 45].

In addition to the opto‐electrical performance of the electrode, the double seed layer improves electron injection in device structures. As confirmed by the EOD results in Supporting Information S1: Figure S6, the Al/MoO_3_ double seed layer exhibits enhanced electron injection compared with the MoO_3_ single seed layer. This improvement is attributed to the formation of a more favorable cathode interface, where the ultrathin Al layer provides a reduced effective electron injection barrier and/or improved interfacial contact owing to its favorable energy alignment with the conduction band minimum (CBM) of ZnMgO. Consequently, the double‐seed strategy preserves the optical advantage of MoO_3_ while incorporating the electron‐injection benefit of Al.

These results demonstrate that the Al/MoO_3_ double seed layer enables the concurrent realization of high optical transmittance, low sheet resistance, and improved electron injection, making it a promising transparent electrode architecture for high‐performance Tr‐QLEDs.

Although the exact transparency requirement depends on the target application, it is widely accepted that an optical transmittance exceeding approximately 50% is required for a material to be perceived as transparent [46]. In Tr‐QLED devices, however, even when the transparent electrode itself satisfies this criterion, the overall device transmittance is inevitably reduced by additional optical losses from the multilayer stack, including surface reflection, absorption, and light scattering (Supporting Information S1: Figure S7). To ensure sufficient transparency at the device level, we therefore adopted a more stringent criterion of ≥ 60% transmittance for the top transparent electrode. Based on this criterion, we selected two electrode configurations for device‐level comparison: MoO_3_ (1 nm)/Ag (10 nm) with a transmittance of 64.31%, and Al (1 nm)/MoO_3_ (1 nm)/Ag (10 nm) with a transmittance of 63.48%. In contrast, Al (1 nm)/Ag (10 nm) (transmittance of 50.07%) was not included in the main device comparison because it did not satisfy our design criterion of ≥ 60% electrode transmittance for highly transparent Tr‐QLEDs. Nevertheless, its device performance was evaluated for comparison, as discussed below.

Figure 4a illustrates the schematic structure of the Tr‐QLED incorporating the Al/MoO_3_ double seed layer. The SEM image of the corresponding Ag electrodes (Supporting Information S1: Figure S8) shows a uniform and well‐interconnected morphology, which is essential for achieving both high conductivity and high optical transparency in ultrathin metal electrodes. The optical transmittance of the complete device was measured, as shown in Figure 4b. The Tr‐QLED with the Al/MoO_3_ double seed layer exhibited an average transmittance of 54.57% in the visible range and 51.8% at 623 nm, exceeding the ∼50% level commonly regarded as the minimum requirement for transparent display applications. The inset photograph shows the pixel device (1.4 mm × 1.4 mm) under operation, exhibiting uniform electroluminescence and clear double‐sided emission. Figure 4c shows the energy‐level diagram of the Tr‐QLED incorporating the double seed layer. As discussed earlier, the introduction of an ultrathin Al layer at the ZnMgO interface is expected to facilitate electron injection, owing to the small energy offset between the Al work function and the CBM of ZnMgO, compared with the ZnMgO/MoO_3_ interface.

**FIGURE 4:**
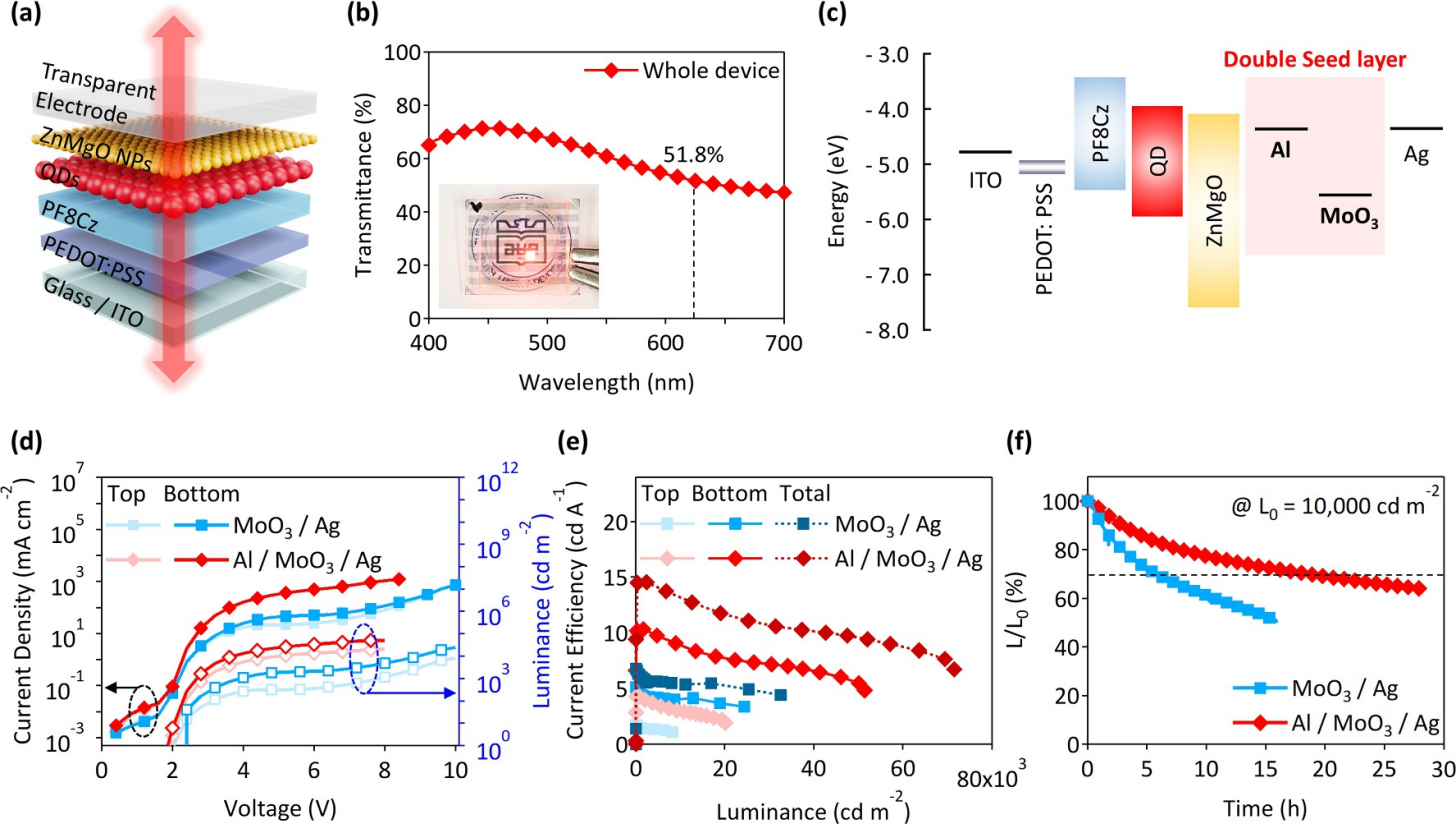
(a) Device schematic of the Tr‐QLED incorporating the Al/MoO_3_ double seed layer and a 10 nm Ag top electrode. (b) Optical transmittance of the Tr‐QLED with the Al/MoO_3_ double seed layer; inset: photograph of a pixel device during operation (substrate size: 20 mm × 24 mm; pixel size: 1.4 mm × 1.4 mm). The dashed line indicates the device transmittance at 623 nm. (c) Energy band diagram of the Tr‐QLED incorporating the Al/MoO_3_ double seed layer. (d) Current density–voltage–luminance (*J–V–L*) characteristics, (e) current efficiency–luminance (CE–*L*) characteristics, and (f) operational lifetime of Tr‐QLEDs with different seed‐layer configurations.

The electrical and optoelectronic characteristics of Tr‐QLEDs incorporating different seed layers are presented in Figure 4d,e. Devices employing the MoO_3_ single seed layer exhibited relatively inefficient electron injection, which resulted in reduced current densities and consequently limited device performance. By contrast, incorporating the Al/MoO_3_ double seed layer improved electron injection and facilitated better charge balance, leading to substantially enhanced device performance.

This improvement is quantitatively summarized in Table 3. When the combined output from both bottom and top emission is considered, the device with the Al/MoO_3_ double seed layer achieved a maximum luminance (*L*_Max_) of 71,554 cd m^−2^ and a maximum current efficiency (CE_Max_) of 14.59 cd A^−1^. In comparison, the device with the MoO_3_ seed layer exhibited significantly lower values of 32,556 cd m^−2^ and 6.78 cd A^−1^, respectively. The EQE‐luminance characteristics are shown in Supporting Information S1: Figure S9: the Al/MoO_3_ double‐seed device reached 9.81%, whereas the MoO_3_ single‐seed device showed a lower value of 4.82%.

**TABLE 3:** Summary of electroluminescence (EL) performance of Tr‐QLEDs, measured from both top and bottom emission directions.

Seed layer	Emission	*L*_Max_ (cd m^−2^)	CE_Max_ (cd A^−1^)	EQE_Max_ (%)
MoO_3_	Top	8238	1.70	1.18
	Bottom	24,318	5.12	3.64
	Total	32,556	6.78	4.82
Al/MoO_3_	Top	20,118	4.28	2.86
	Bottom	51,436	10.31	6.95
	Total	71,554	14.59	9.81

To provide a more comprehensive comparison, the device characteristics and performance of Tr‐QLEDs employing the Al/Ag electrode are presented in Supporting Information S1: Figure S10 and summarized in Supporting Information S1: Table S1. Although the Al/Ag device exhibited a higher maximum bottom‐emission luminance than the Al/MoO_3_/Ag double‐seed device, this difference may be related to the distinct optical transmittance and reflectance of the two top electrodes, resulting in different directional light‐outcoupling characteristics. In contrast, the top‐emission luminance of the Al/Ag device was lower, consistent with the substantially lower optical transmittance of the Al/Ag electrode (50.07%) compared with that of the Al/MoO_3_/Ag electrode (63.48%). In addition, the Al/Ag device exhibited lower current efficiency and external quantum efficiency (CE = 11.33 cd A^−1^, EQE = 8.06%) than the Al/MoO_3_ double‐seed device (CE = 14.59 cd A^−1^, EQE = 9.81%). These results indicate that the Al/MoO_3_ double‐seed architecture provides higher top‐side light extraction and superior overall efficiency, resulting in a more balanced optical output for Tr‐QLED operation.

Although both bottom and top emission were clearly observed, the top‐emission efficiency was intrinsically lower. This reduction can be attributed to the lower optical transmittance of the top Ag electrode compared with the bottom ITO electrode, together with additional optical losses introduced by the encapsulation glass. Consequently, a fraction of emitted photons is absorbed or reflected at the top electrode and the encapsulation layers, whereas light extracted through the highly transparent bottom ITO electrode experiences fewer optical losses. This asymmetry represents a remaining limitation of the current architecture. Therefore, in addition to further improving the transparency of the top electrode, future efforts should focus on reducing optical losses associated with the encapsulation structure through optimized encapsulation designs and optical capping layers to further enhance top‐emission efficiency.

Device operational stability was evaluated under constant‐current operation at an initial luminance of 10,000 cd m^−2^ (Figure 4f). The Tr‐QLED with the Al/MoO_3_ double seed layer exhibited a *T*_70_ lifetime of 18.56 h, which is more than three times longer than the 5.81 h recorded for the MoO_3_‐only device. Using the commonly employed acceleration relation *L*_0_^*n*^ *T*_70_ = constant with *n* = 1.8, which has been widely adopted for lifetime extrapolation in previous QD‐LED and OLED studies [8, 47, 48, 49], these values correspond to extrapolated *T*_70_ lifetimes of 73,888 h and 23,130 h at 100 cd m^−2^, respectively. This substantial improvement in operational stability can be attributed to the contrasting charge injection characteristics of the two electrode structures. In the MoO_3_‐only device, inefficient electron injection likely leads to charge imbalance, which can enhance non‐radiative loss channels (e.g., Auger‐related processes and interfacial quenching) and accelerate device degradation. In contrast, the Al/MoO_3_ double seed layer promotes more balanced charge injection, thereby suppressing these loss processes and enabling more stable operation over extended periods.

The transparent electrode properties and device performance of the present Tr‐QLEDs are further benchmarked against previously reported Tr‐QLEDs employing various transparent electrode technologies in Supporting Information S1: Tables S2 and S3. While some previous studies reported higher optical transmittance or device efficiency, the proposed Al/MoO_3_/Ag electrode combines a notably low sheet resistance with relatively high luminance using a simple sequential thermal evaporation process. Such a process is well suited for the uniform deposition of transparent electrodes over large areas, which is an important requirement for practical display manufacturing.

To further validate the scalability of the proposed approach, we fabricated large‐area Tr‐QLEDs on a 2 inch × 2 inch substrate with an 8 × 8 pixel array, where each pixel has a size of 3 mm × 3 mm (Figure 5). Figure 5a,b show the device in the off state under outdoor conditions, where the background scenery is clearly visible through the device, demonstrating high optical transparency. Figure 5c presents the off‐state device under indoor conditions, further confirming the transparency of the large‐area structure. Upon operation (Figure 5d), the device exhibits uniform electroluminescence across the entire pixel array, indicating consistent pixel performance and stable large‐area emission. An enlarged photograph of the corresponding emission region is provided in Figure 5e, further confirming the uniform electroluminescence of the large‐area Tr‐QLEDs.

**FIGURE 5:**
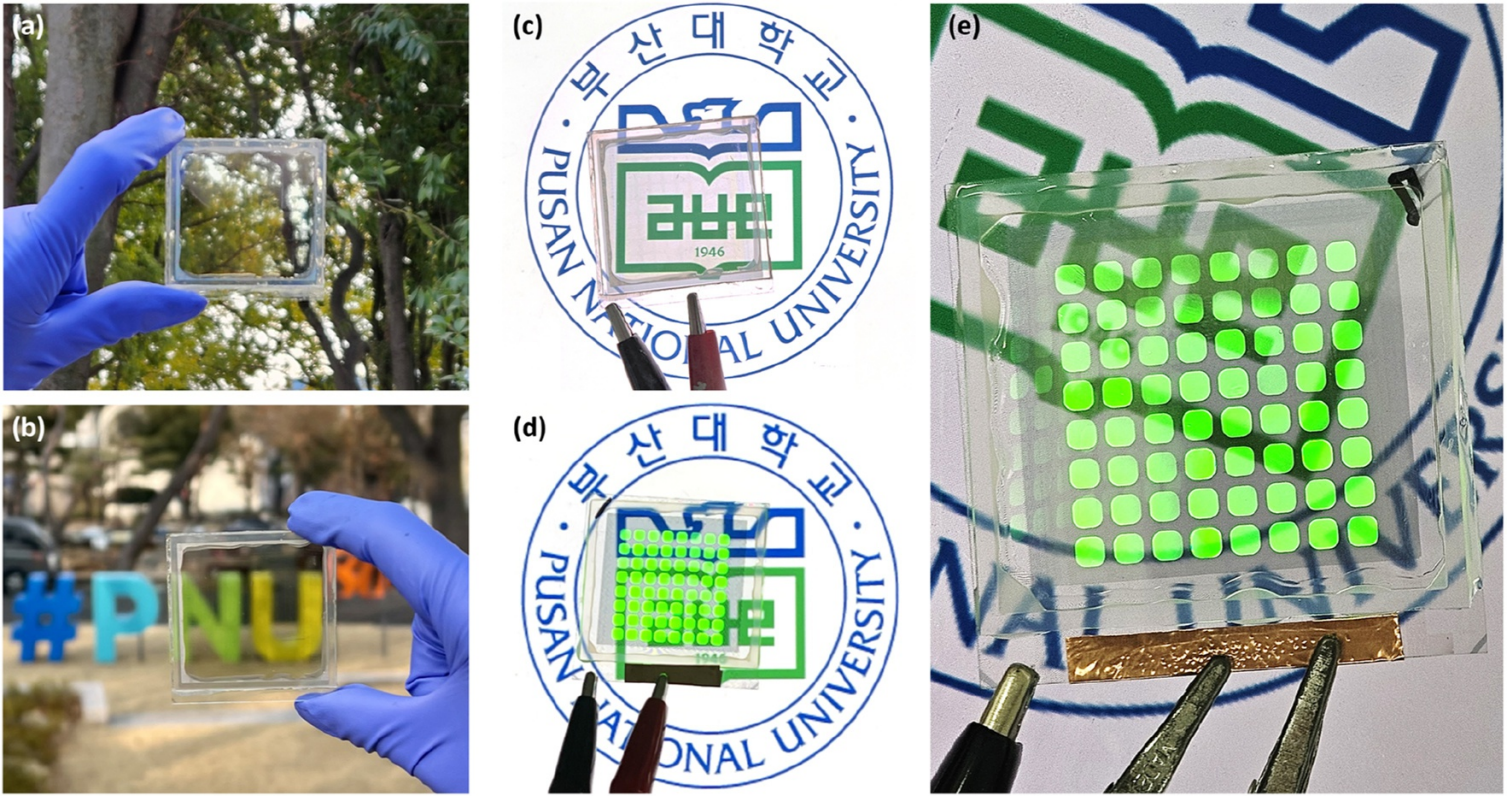
Photographs of large‐area Tr‐QLEDs incorporating the Al/MoO_3_ double‐seed electrode (substrate size: 2 inch × 2 inch; 8 × 8 pixel array; pixel size: 3 mm × 3 mm). (a, b) Devices in the off state under outdoor conditions, clearly showing background visibility through the transparent device. (c) Device in the off state under indoor conditions. (d) Device in operation under indoor conditions, exhibiting uniform electroluminescence. (e) Enlarged photograph of the electroluminescence emission region of the large‐area Tr‐QLED shown in Panel (d).

As discussed in the introduction, thermally evaporated Ag films are well‐suited for large‐area transparent electrodes because vacuum deposition enables uniform thin‐film formation with a simple and scalable process. The successful demonstration of the large‐area device underscores the compatibility of this strategy with practical display manufacturing.

Taken together, these results demonstrate that the Al/MoO_3_ double seed layer enables efficient charge injection and high device performance while maintaining device‐level transparency, offering a viable pathway toward scalable and manufacturable Tr‐QLEDs.

## 3 Conclusion

In summary, we demonstrated Tr‐QLEDs employing an Al/MoO_3_ double seed layer that enabled the formation of ultrathin Ag electrodes with enhanced performance. For continuous thin Ag film formation, seed layers are essential. While MoO_3_ provides excellent transmittance and low sheet resistance, it suffers from limited electron injection. In contrast, Al improves electron injection owing to its favorable energy‐level alignment, despite its lower optical transmittance. The double seed layer strategy effectively combines these complementary advantages, thereby overcoming the limitations of single seed layer approaches. This double seed layer design enables continuous Ag growth at a thickness of 10 nm, simultaneously achieving high transparency, low resistance, and efficient charge injection. Tr‐QLEDs incorporating the double seed layer exhibited over twofold higher luminance and EQE and threefold longer operational lifetime compared with MoO_3_ single seed layer devices. Furthermore, large‐area (2 inch × 2 inch) Tr‐QLEDs were fabricated, thereby confirming the scalability of this approach. These findings establish the Al/MoO_3_ double seed layer as a practical and effective strategy for transparent electrodes in QD‐LEDs. By resolving the long‐standing trade‐off in ultrathin Ag films, this work provides a reliable pathway toward high‐performance, large‐area, and double‐sided emitting Tr‐QLEDs, highlighting their strong potential for commercialization in next‐generation transparent display and lighting technologies.

## 4 Experimental Section/Methods

Materials: Pre‐patterned ITO‐coated glass substrates were purchased from AMG. Zinc acetate dihydrate (99%), magnesium acetate tetrahydrate (98%), tetramethylammonium hydroxide (TMAH, 97%), ethanol (anhydrous, 99.5%), chlorobenzene (anhydrous, 99.8%), octane (99%), zinc acetate (Zn(OAc)2, 99.99%), cadmium oxide (CdO, > 99.99%), trioctylphosphine (TOP, 97%), selenium (Se, 99.99%), sulfur (S, 99.99%), oleic acid (OA, 90%), 1‐octadecene (ODE, 90%), and cyclohexane (anhydrous, 97%) were purchased from Sigma‐Aldrich. Acetone (99.7%), isopropanol (IPA; 99.9%), ethyl acetate (99.7%), and dimethyl sulfoxide (DMSO; 99.9%) were purchased from SAMCHUN Chemicals. PEDOT:PSS (Clevios P VP AI 4083) was purchased from the Heraeus Electronic Materials division. Poly[(9,9‐dioctylfluorenyl‐2,7‐diyl)‐alt‐(9‐(2‐ethylhexyl)‐carbazole‐3,6‐diyl)] (PF8Cz) was obtained from OSM. Molybdenum trioxide (MoO_3_, 99.995%) powders, aluminum (Al, 99.999%) metal pellets and silver (Ag, 99.99%) granules were purchased from Taewon Scientific (iTASCO).

Synthesis of red‐emitting colloidal QDs: Red‐emitting colloidal core/shell QDs were prepared following modified procedures based on previously reported methods [50, 51]. For the synthesis of CdZnSe/ZnSe/ZnSeS/ZnS QDs, 1.4 mmol of CdO and 1.8 mmol of Zn(OAc)_2_ were mixed with 10.0 mL of OA and 20.0 mL of ODE. The mixture was heated at 120°C under vacuum for 1 h to generate Cd‐ and Zn‐oleate complexes. After the complex formation step, the reaction temperature was increased to 310°C under an Ar atmosphere. A solution of TOP‐Se (0.5 M, 6.0 mL) was then rapidly injected to initiate nucleation and growth of CdZnSe cores. Following 1 h of growth, a ZnSe shell was formed by injecting 4.0 mL of Zn(OAc)_2_ solution (0.4 M) together with 4.0 mL of TOP‐Se (0.5 M), followed by an additional 20 min of reaction. Subsequently, the ZnSeS intermediate shell was formed through the gradual addition of 4.0 mL Zn(OAc)_2_ (0.4 M), 2.0 mL TOP‐Se (0.5 M), and 2.0 mL TOP‐S (0.5 M). After 20 min, 4.0 mL of Zn(OAc)_2_ solution (0.4 M) together with 4.0 mL of TOP‐S (0.5 M) were slowly injected for the growth of the outer ZnS shell layer, followed by an additional 20 min of reaction. The products were purified using cyclohexane and acetone. After purification, the final red‐emitting QDs were dispersed in cyclohexane for subsequent use.

Synthesis of Zn_0.85_Mg_0.15_O nanoparticles: Zn_0.85_Mg_0.15_O nanoparticles (NPs) were synthesized by dissolving zinc acetate dihydrate and magnesium acetate tetrahydrate in DMSO, followed by stirring at 500 rpm under ambient conditions for several minutes. A pre‐sonicated TMAH solution in ethanol was then added into the mixture under continuous stirring. The reaction was maintained for 45 min at a constant temperature to complete the synthesis. The obtained ZnMgO NPs were purified by centrifuging three times with ethyl acetate and ethanol. Finally, the purified ZnMgO NPs were redispersed in ethanol for subsequent use in QD‐LED fabrication.

Device fabrication: Pre‐patterned ITO‐coated glass substrates were sequentially cleaned using acetone, IPA, and deionized water via ultrasonication for 15 min each. The cleaned substrates were dried with N_2_ and baked overnight in an oven to remove residual moisture. Prior to deposition, the substrates were exposed to ultraviolet ozone treatment for 15 min to increase the hydrophilicity of the ITO surface and to remove any remaining organic contaminants, thereby facilitating uniform film formation. For the Tr‐QLEDs (structure: ITO/PEDOT:PSS/PF8Cz/QD/ZnMgO/transparent electrode/encapsulation), PEDOT:PSS was then spin‐coated at 3000 rpm for 45 s and subsequently baked at 150°C for 15 min. The PEDOT:PSS‐coated substrates were transferred into a N_2_‐filled glove box for the deposition of the following layers. PF8Cz solution (8 mg mL^−1^ in chlorobenzene) was spin‐coated at 4000 rpm for 30 s and annealed at 150°C for 30 min. Next, QD solution (15 mg mL^−1^ in octane) was spin‐coated at 4000 rpm for 30 s and baked at 90°C for 30 min. Finally, a solution of ZnMgO NPs (20 mg mL^−1^ in ethanol) was spin‐coated at 2000 rpm for 45 s and annealed at 90°C for 30 min. For the EOD devices (structure: ITO/ZnMgO/QD/ZnMgO/transparent electrode/encapsulation), the fabrication procedure was similar, except that the bottom PF8Cz and PEDOT:PSS layers were omitted and the QD layer was directly deposited on the ZnMgO‐coated ITO. After completing all solution‐processed layers, the substrates were transferred to a thermal evaporation chamber, where Al, MoO_3_, and Ag layers were sequentially deposited at a pressure below 10^−6^ torr to form the transparent top electrode. The deposition rates for Al and MoO_3_ were 0.2 Å s^−1^, whereas Ag was deposited at 1.0 Å s^−1^. All devices were subsequently encapsulated with an ultraviolet (UV)‐curing resin and cover glass.

Film and device characterization: Scanning electron microscopy (SEM) images were acquired using a GEMINI500 system (ZEISS). Optical transmittance spectra were measured with a V‐730 UV‐visible and NIR spectrometer (JASCO Instruments). Sheet resistance values were obtained by averaging measurements at three different positions on each sample using a resistivity meter (DASOLENG). The thicknesses of the ultrathin electrode stacks were characterized by step‐height measurements using an NX10 atomic force microscope (AFM, Park Systems). Ultraviolet photoelectron spectroscopy (UPS) measurements were performed using an AXIS SUPRA system (Kratos Analytical). Current density–voltage–luminance (*J–V–L*) and external quantum efficiency–luminance (EQE*–L*) characteristics were recorded with a Keithley 2400 source meter (Tektronix) and a spectroradiometer CS‐2000 (Konica Minolta). The operational lifetime of the devices was evaluated using an M6000 PLUS lifetime measurement system (McScience).

## Author Contributions

**Yeyun Bae:** conceptualization, methodology, writing – original draft. **Kyoungeun Lee:** conceptualization, methodology, writing – original draft. **Kyunghoon Lee:** methodology, resources. **Jaeyeop Lee:** validation. **Jiyoon Oh:** validation. **Jiwoong Yang:** supervision, resources. **Chansu Kim:** resources, supervision, validation. **Jeongkyun Roh:** writing – review and editing, supervision, validation.

## Supporting information


Supporting Information S1

## Data Availability

The data that support the findings of this study are available from the corresponding author upon reasonable request.

## References

[1] Z. Tan , F. Zhang , T. Zhu , et al., “Bright and Color‐Saturated Emission From Blue Light‐Emitting Diodes Based on Solution‐Processed Colloidal Nanocrystal Quantum Dots,” Nano Letters 7, no. 12 (2007): 3803–3807, 10.1021/nl072370s. 17975946

[2] O. Chen , J. Zhao , V. P. Chauhan , et al., “Compact High‐Quality CdSe–CdS Core–Shell Nanocrystals With Narrow Emission Linewidths and suppressed blinking,” Nature Materials 12, no. 5 (2013): 445–451, 10.1038/nmat3539. PMC3677691 23377294

[3] L. Qian , Y. Zheng , J. Xue , and P. H. Holloway , “Stable and Efficient Quantum‐Dot Light‐Emitting Diodes Based on Solution‐Processed Multilayer Structures,” Nature Photonics 5, no. 9 (2011): 543–548, 10.1038/nphoton.2011.171.

[4] M. D. Tessier , D. Dupont , K. De Nolf , J. De Roo , and Z. Hens , “Economic and Size‐Tunable Synthesis of InP/ZnE (E = S, Se) Colloidal Quantum Dots,” Chemistry of Materials 27, no. 13 (2015): 4893–4898, 10.1021/acs.chemmater.5b02138.

[5] J. Kwak , J. Lim , M. Park , S. Lee , K. Char , and C. Lee , “High‐Power Genuine Ultraviolet Light‐Emitting Diodes Based On Colloidal Nanocrystal Quantum Dots,” Nano Letters 15, no. 6 (2015): 3793–3799, 10.1021/acs.nanolett.5b00392. 25961530

[6] J. Kim , J. Roh , M. Park , and C. Lee , “Recent Advances and Challenges of Colloidal Quantum Dot Light‐Emitting Diodes for Display Applications,” Advanced Materials 36, no. 20 (2024): 2212220, 10.1002/adma.202212220. 36853911

[7] J. Lee , H. Jo , M. Choi , et al., “Recent Progress on Quantum Dot Patterning Technologies for Commercialization of QD‐LEDs: Current Status, Future Prospects, and Exploratory Approaches,” Small Methods 8, no. 7 (2024): 2301224, 10.1002/smtd.202301224. 38193264

[8] T. Davidson‐Hall and H. Aziz , “Perspective: Toward Highly Stable Electroluminescent Quantum Dot Light‐Emitting Devices in the Visible Range,” Applied Physics Letters 116, no. 1 (2020): 010502, 10.1063/1.5134090.

[9] S. Rhee , K. Kim , J. Roh , and J. Kwak , “Recent Progress in High‐Luminance Quantum Dot Light‐Emitting Diodes,” Current Optics and Photonics 4, no. 3 (2020): 161–173, 10.3807/COPP.2020.4.3.161.

[10] H. Seo , G. Park , B. Chun , et al., “A Comprehensive Review on the Lifetime of Cadmium‐Free Quantum‐Dot Light‐Emitting Diodes,” Journal of Infectious Diseases 26, no. 4 (2025): 381–397, 10.1080/15980316.2025.2500618.

[11] J. Lee , S. L. Kwak , C. Lim , et al., “Universal and Nondestructive Direct Photolithography of Colloidal Quantum Dots Using Photocrosslinkable Polymer Blends,” Advances in Functional Materials 36, no. 10 (2026): e20047, 10.1002/adfm.202520047.

[12] Y. Bae , J. Lee , K. Lee , et al., “Charge Generation Junction for Efficient Hole Injection in InP‐Based Quantum Dot Light‐Emitting Diodes,” ACS Applied Electronic Materials 7, no. 10 (2025): 4493–4500, 10.1021/acsaelm.5c00342.

[13] K. Lee , J. Lee , Y. Bae , et al., “Interfacial Modification of ZnO/ZnMgO Bilayer for Efficient and Stable InP Quantum Dot Light‐Emitting Diodes via Ultraviolet Ozone Treatment,” ACS Applied Materials & Interfaces 16, no. 41 (2024): 55658–55665, 10.1021/acsami.4c09900. 39374374

[14] J. Lee , Y. Bae , K. Lee , et al., “Positive Aging‐Free Quantum Dot Light‐Emitting Diodes Enabled by Single‐Source Chloride‐Doped ZnMgO Electron Transport Layers,” Small 22, no. 37 (2026): e14995, 10.1002/smll.202514995. PMC13325696 42117282

[15] J. Lee , S. L. Kwak , C. Lim , et al., “Universal and Nondestructive Direct Photolithography of Colloidal Quantum Dots Using Photocrosslinkable Polymer Blends,” Advances in Functional Materials 36, no. 10 (2026): e20047, 10.1002/adfm.202520047.

[16] N. Zhang , X. Qu , Q. Lyu , K. Wang , and X. W. Sun , “Highly Efficient Transparent Quantum‐Dot Light‐Emitting Diodes Based on Inorganic Double Electron‐Transport Layers,” Photonics Research 9, no. 10 (2021): 1979–1983, 10.1364/prj.424750.

[17] M. K. Choi , J. Yang , D. C. Kim , et al., “Extremely Vivid, Highly Transparent, and Ultrathin Quantum Dot Light‐Emitting Diodes,” Advanced Materials 30, no. 1 (2018): 1703279, 10.1002/adma.201703279. 29068560

[18] H. Li , S. Zhou , and S. Chen , “Highly Efficient Top‐Emitting Quantum‐Dot Light‐Emitting Diodes With Record‐Breaking External Quantum Efficiency of Over 44.5%,” Laser & Photonics Reviews 17, no. 8 (2023): 2300371, 10.1002/lpor.202300371.

[19] P. Jing , W. Ji , Q. Zeng , et al., “Vacuum‐Free Transparent Quantum Dot Light‐Emitting Diodes With Silver Nanowire Cathode,” Scientific Reports 5, no. 1 (2015): 12499, 10.1038/srep12499. PMC4510483 26198668

[20] Y. Yu , Y. Liang , J. Yong , et al., “Low‐Temperature Solution‐Processed Transparent QLED Using Inorganic Metal Oxide Carrier Transport Layers,” Advances in Functional Materials 32, no. 3 (2022): 2106387, 10.1002/adfm.202106387.

[21] S. Kim , J. Kim , D. Kim , et al., “High‐Performance Transparent Quantum Dot Light‐Emitting Diode With Patchable Transparent Electrodes,” ACS Applied Materials & Interfaces 11, no. 29 (2019): 26333–26338, 10.1021/acsami.9b05969. 31286764

[22] Z. Yu , L. Hu , Z. Liu , et al., “Enhancing the Photocurrent in Poly(3‐Hexylthiophene)/[6,6]‐Phenyl C_61_ Butyric Acid Ethyl Ester Bulk Heterojunction Solar Cells by Using Poly(3‐Hexylthiophene) as a Buffer Layer,” Applied Physics Letters 95, no. 20 (2009): 133303, 10.1063/1.3242006.

[23] Z. Li , P. P. Boix , G. Xing , et al., “Carbon Nanotubes as an Efficient Hole Collector for High Voltage Methylammonium Lead Bromide Perovskite Solar Cells,” Nanoscale 8, no. 12 (2016): 6352–6360, 10.1039/c5nr06177f. 26646241

[24] J. Ouyang , “‘Secondary Doping’ Methods to Significantly Enhance the Conductivity of PEDOT: PSS for Its Application as Transparent Electrode of Optoelectronic Devices,” Displays 34, no. 5 (2013): 423–436, 10.1016/j.displa.2013.08.007.

[25] M. Vosgueritchian , D. J. Lipomi , and Z. Bao , “Highly Conductive and Transparent PEDOT: PSS Films With a Fluorosurfactant for Stretchable and Flexible Transparent Electrodes,” Advances in Functional Materials 22, no. 2 (2012): 421–428, 10.1002/adfm.201101775.

[26] L. Yao , X. Fang , W. Gu , et al., “Fully Transparent Quantum Dot Light‐Emitting Diode With a Laminated Top Graphene Anode,” ACS Applied Materials & Interfaces 9, no. 28 (2017): 24005–24010, 10.1021/acsami.7b02026. 28631480

[27] J.‐H. Chang , W.‐H. Lin , P.‐C. Wang , et al., “Solution‐Processed Transparent Blue Organic Light‐Emitting Diodes With Graphene as the Top Cathode,” Scientific Reports 5, no. 1 (2015): 9693, 10.1038/srep09693. PMC4402614 25892370

[28] F. Fries , M. Fröbel , S. Lenk , and S. Reineke , “Transparent and Color‐Tunable Organic Light‐Emitting Diodes With Highly Balanced Emission to Both Sides,” Organic Electronics 41 (2017): 315–318, 10.1016/j.orgel.2016.11.022.

[29] J. Zou , C. Z. Li , C. Y. Chang , H. L. Yip , and A. K. Y. Jen , “Interfacial Engineering of Ultrathin Metal Film Transparent Electrode for Flexible Organic Photovoltaic Cells,” Advanced Materials 26, no. 22 (2014): 3618–3623, 10.1002/adma.201306212. 24623553

[30] X. Yang , P. Gao , Z. Yang , J. Zhu , F. Huang , and J. Ye , “Optimizing Ultrathin Ag Films for High Performance Oxide‐Metal‐Oxide Flexible Transparent Electrodes Through Surface Energy Modulation and Template‐Stripping Procedures,” Scientific Reports 7, no. 1 (2017): 44576, 10.1038/srep44576. PMC5349598 28291229

[31] H. Wei and H. Eilers , “From Silver Nanoparticles to Thin Films: Evolution of Microstructure and Electrical Conduction on Glass Substrates,” Journal of Physics and Chemistry of Solids 70, no. 2 (2009): 459–465, 10.1016/j.jpcs.2008.11.012.

[32] S. Yang and H. S. Chen , “Highly Efficient Double‐Side‐Emitting Electroluminescent Quantum Dot Thin Film With Transparent MoO3/Ag:Cu/MoO3 Electrode Prepared by Thermal Co‐Evaporation,” Advanced Materials Technologies 8, no. 2 (2023): 2201077, 10.1002/admt.202201077.

[33] T.‐H. Yeh , C.‐C. Lee , C.‐J. Shih , G. Kumar , S. Biring , and S.‐W. Liu , “Vacuum‐Deposited MoO3/Ag/WO3 Multilayered Electrode for Highly Efficient Transparent and Inverted Organic Light‐Emitting Diodes,” Organic Electronics 59 (2018): 266–271, 10.1016/j.orgel.2018.05.014.

[34] T. Schwab , S. Schubert , L. Müller‐Meskamp , K. Leo , and M. C. Gather , “Eliminating Micro‐Cavity Effects in White Top‐Emitting OLEDs by Ultra‐Thin Metallic Top Electrodes,” Advanced Optical Materials 1, no. 12 (2013): 921–925, 10.1002/adom.201300392.

[35] J. H. Im , K.‐T. Kang , S. H. Lee , J. Y. Hwang , H. Kang , and K. H. Cho , “Bulk‐Like Al/Ag Bilayer Film Due To Suppression of Surface Plasmon Resonance for High Transparent Organic Light Emitting Diodes,” Organic Electronics 33 (2016): 116–120, 10.1016/j.orgel.2016.03.002.

[36] Q. Wan , C. Yuan , X. Liao , et al., “Semitransparent Light‐Emitting Diodes Based on InP/ZnSeS/ZnS Quantum Dots With Al/Ag Double Metal Layer as Top Electrode,” Journal of Information Display 26, no. 2 (2025): 87–94, 10.1080/15980316.2025.2495966.

[37] N. Kaiser , “Review of the Fundamentals of Thin‐Film Growth,” Applied Optics 41, no. 16 (2002): 3053–3060, 10.1364/ao.41.003053. 12064380

[38] D. Li , Y. Pan , H. Liu , Y. Zhang , Z. Zheng , and F. Zhang , “Study on Ultrathin Silver Film Transparent Electrodes Based on Aluminum Seed Layers With Different Structures,” Nanomaterials 12, no. 19 (2022): 3540, 10.3390/nano12193540. PMC9565528 36234666

[39] C. Zhang , C. Ji , Y. B. Park , and L. J. Guo , “Thin‐Metal‐Film‐Based Transparent Conductors: Material Preparation, Optical Design, and Device Applications,” Advanced Optical Materials 9, no. 3 (2021): 2001298, 10.1002/adom.202001298.

[40] S. Schubert , J. Meiss , L. Müller‐Meskamp , and K. Leo , “Improvement of Transparent Metal Top Electrodes for Organic Solar Cells by Introducing a High Surface Energy Seed Layer,” Advanced Energy Materials 3, no. 4 (2013): 438–443, 10.1002/aenm.201200903.

[41] X. Zhang , Q. Zhang , T. Yan , Z. Jiang , X. Zhang , and Y. Y. Zuo , “Quantitatively Predicting Bacterial Adhesion Using Surface Free Energy Determined With a Spectrophotometric Method,” Environmental Science & Technology 49, no. 10 (2015): 6164–6171, 10.1021/es5050425. PMC4854535 25898026

[42] A. Toikka , M. Ilin , and N. Kamanina , “Perspective Coatings Based on Structured Conducting ITO Thin Films for General Optoelectronic Applications,” Coatings 14, no. 2 (2024): 178, 10.3390/coatings14020178.

[43] C. Ji , D. Liu , C. Zhang , and L. Jay Guo , “Ultrathin‐Metal‐Film‐Based Transparent Electrodes With Relative Transmittance Surpassing 100%,” Nature Communications 11, no. 1 (2020): 3367, 10.1038/s41467-020-17107-6. PMC7338390 32632111

[44] G. Perrakis , A. C. Tasolamprou , G. Kakavelakis , et al., “Infrared‐Reflective Ultrathin‐Metal‐Film‐Based Transparent Electrode With Ultralow Optical Loss for High Efficiency in Solar Cells,” Scientific Reports 14, no. 1 (2024): 548, 10.1038/s41598-023-50988-3. PMC10766980 38177236

[45] B. Zhang , R. Gong , Y. Zhang , Y. Li , and L. Zhu , “Recent Progress in Dielectric/Metal/Dielectric Electrodes for Foldable Light‐Emitting Devices,” Nanotechnology Reviews 12, no. 1 (2023): 20230578, 10.1515/ntrev-2023-0578.

[46] H. Quan , W. Shi , and L. Kong , “Non‐Destructive Optical Measurement of Transparent Objects: A Review,” Light: Advanced Manufacturing 6 (2025): 1–25, 10.37188/lam.2025.022.

[47] M. Azadinia , P. Chun , Q. Lyu , G. Cotella , and H. Aziz , “Differences in Electron and Hole Injection and Auger Recombination Between Red, Green, and Blue CdSe‐Based Quantum Dot Light Emitting Devices,” ACS Nano 18, no. 2 (2024): 1485–1495, 10.1021/acsnano.3c07999. 38175971

[48] M. Azadinia , T. Davidson‐Hall , D. S. Chung , et al., “Inverted Solution‐Processed Quantum Dot Light‐Emitting Devices With Wide Band Gap Quantum Dot Interlayers,” ACS Applied Materials & Interfaces 15, no. 19 (2023): 23631–23641, 10.1021/acsami.3c02356. 37141421

[49] W. Cao , C. Xiang , Y. Yang , et al., “Highly Stable QLEDs With Improved Hole Injection via Quantum dot Structure Tailoring,” Nature Communications 9, no. 1 (2018): 2608, 10.1038/s41467-018-04986-z. PMC6031613 29973590

[50] J. Yoo , K. Lee , U. J. Yang , et al., “Highly Efficient Printed Quantum Dot Light‐Emitting Diodes Through Ultrahigh‐Definition Double‐Layer Transfer Printing,” Nature Photonics 18, no. 10 (2024): 1105–1112, 10.1038/s41566-024-01496-x.

[51] J. Lee , Y. Kim , K. Lee , et al., “Transfer‐Printed Multi‐Stacked Quantum Dot Color Conversion Layers for White Light‐Emitting Diodes,” Applied Surface Science 687 (2025): 162196, 10.1016/j.apsusc.2024.162196.

