# Supplementary material for: Decoupling Transparency and Electron Injection in Ultrathin Ag Electrodes Using an Al/MoO3 Double Seed Layer for Transparent Quantum Dot Light‐Emitting Diodes
Source: Nanophotonics. 2026 Sep 3;15(17):e70283. doi: 10.1002/nap2.70283 (PMC13672127; doi:10.1002/nap2.70283)
Supplement: Supplementary file 1 [file NAP2-15-e70283-s001.docx]

Supporting Information

Decoupling Transparency and Electron Injection in Ultrathin Ag Electrodes Using an Al/MoO_3_ Double Seed Layer for Transparent Quantum Dot Light-Emitting Diodes

Yeyun Bae, Kyoungeun Lee, Kyunghoon Lee, Jaeyeop Lee, Jiyoon Oh, Jiwoong Yang, Chansu Kim*, and Jeongkyun Roh*

Figure S1. Optical transmittance spectra of Ag thin films with different thicknesses (8, 10, and 15 nm) deposited on (a) bare glass, (b) Al (1 nm), (c) MoO_3_ (1 nm), and (d) Al/MoO_3_ (1 nm/1 nm) seed layers.

Figure S2. Scanning electron microscopy (SEM) images of 10 nm Ag films deposited on (a) bare glass, (b) Al (1 nm), and (c) MoO_3_ (1 nm) seed layers. The Ag film without a seed layer exhibits a discontinuous island-like morphology, whereas the Al or MoO_3_ seed layer promotes a continuous film. The inset schematics illustrate the corresponding growth morphology for each case.

Figure S3. Ultraviolet photoelectron spectroscopy (UPS) spectra of the ZnMgO/electrode stacks with 10 nm Ag: ZnMgO/Ag, ZnMgO/Al/Ag, and ZnMgO/MoO_3_/Ag. (a) Secondary electron cutoff spectra and (b) near-Fermi-level spectra. UPS measurements were performed from the top Ag surface.

Figure S4. Atomic force microscopy (AFM) step-height profiles of (a) Al (1 nm)/Ag (10 nm), (b) MoO_3_ (1 nm)/Ag (10 nm), and (c) Al (1 nm)/MoO_3_ (1 nm)/Ag (10 nm) electrode stacks deposited on glass substrates. The measured step heights are in the range of approximately 11–12.5 nm, consistent with the expected nominal thicknesses of the ultrathin electrode stacks.

Figure S5. Optical transmittance and reflectance spectra of (a) Ag (10 nm), (b) Al (1 nm)/Ag (10 nm), (c) MoO_3_ (1 nm)/Ag (10 nm), and (d) Al (1 nm)/MoO_3_ (1 nm)/Ag (10 nm) electrodes deposited on glass substrates. The dashed lines indicate the wavelength (623 nm) used for comparison of the optical properties. Insets show photographs of the corresponding transparent electrodes.

Figure S6. Current density–voltage (*J–V*) characteristics of electron-only devices (EODs) with different seed layer configurations: Al (1 nm)/Ag (10 nm), MoO_3_ (1 nm)/Ag (10 nm), and Al (1 nm)/MoO_3_ (1 nm)/Ag (10 nm).

Figure S7. Schematic illustration of optical loss mechanisms in (a) a single-layer system and (b) a multilayer Tr-QLED structure. Incident light (I_0_) is partially reflected (I_r_), absorbed (I_a_), or scattered (I_s_), reducing the transmitted light (I_t_).

Figure S8. An SEM image of 10 nm Ag film deposited on the Al/MoO_3_ double seed layer. The inset schematic illustrates the corresponding growth morphology.

Figure S9. External quantum efficiency–luminance (EQE–*L*) characteristics of Tr-QLEDs with different seed layer configurations.

Figure S10. (a) Current density–voltage–luminance (*J*–*V*–*L*) characteristics, (b) current efficiency–luminance (CE–*L*) characteristics, and (c) external quantum efficiency–luminance (EQE–*L*) characteristics of an Al-seeded Tr-QLED.

Table S1. Summary of electroluminescence (EL) performance of an Al-seeded Tr-QLED measured from both top and bottom emission directions.

| Seed layer | Emission | *L*_Max_  (cd m^−2^) | CE_Max_  (cd A^−1^) | EQE_Max_  (%) |
| --- | --- | --- | --- | --- |
| Al | Top | 19,435 | 2.57 | 1.72 |
|  | Bottom | 73,602 | 8.76 | 6.34 |
|  | Total | 93,037 | 11.33 | 8.06 |

Table S2. Comparison of transparent electrode properties of previously reported Tr-QLEDs.

| Transparent electrode | Deposition  method | Reported transmittance  (%) | Rs  ($\Omega$ sq^−1^) | Refs |
| --- | --- | --- | --- | --- |
| Graphene | Transfer | Device: 70-80% | 110 / 741 | (S1) |
| Graphene | Lamination | Electrode: 79.4%  @622 nm | ~540 | (S2) |
| AgNW | Spin coating | Device: >60% | 7.51 | (S3) |
| AgNW | Lamination | Electrode: 70.1%  @550 nm | 7.9 | (S4) |
| ITO | Sputtering | Device: 82%  @550 nm | - | (S5) |
| MXene/AgNW | Spray coating | Electrode: 83.8%  @550 nm | 13.9 | (S6) |
| MXene/AgNW | Mayer bar coating | Electrode: 84.6%  @620 nm | 16.07 | (S7) |
| **Al/MoO_3_/Ag** | **Thermal evaporation** | **Electrode: 63.48%**  **@623 nm** | **7.35** | **This work** |

Table S3. Comparison of device performance of previously reported Tr-QLEDs.

| Transparent electrode | *L*_Max_ (cd m^−2^) | | | CE_Max_ (cd A^−1^) | | | Refs |
| --- | --- | --- | --- | --- | --- | --- | --- |
|  | Top | Bottom | Total | Top | Bottom | Total |  |
| Graphene | 358 ^a)^ | | | 0.45 ^a)^ | | | (S1) |
| Graphene | 12 | 30 | 42 | 0.28 | 0.32 | 0.60 | (S2) |
| AgNW | 23,440 | 25,040 | - | 5.2 | 5.6 | - | (S3) |
| AgNW | 14,902 | 12,408 | 27,310 | 18.17 | 27.82 | 45.99 | (S4) |
| ITO | 33,764 ^a)^ | | | 16 ^a)^ | | | (S5) |
| MXene/AgNW | 31,340 ^a)^ | | | 25.8 ^a)^ | | | (S6) |
| MXene/AgNW | 9,008 | 12,007 | 21,015 | 9.20 | 13.92 | 23.12 | (S7) |
| **Al/MoO_3_/Ag** | **20,118** | **51,436** | **71,554** | **4.28** | **10.31** | **14.59** | **This work** |

^a)^ The original publication did not specify whether the reported value corresponds to top, bottom, or total emission.

REFERENCES

S1.	Seo, J.-T.; Han, J.; Lim, T.; Lee, K.-H.; Hwang, J.; Yang, H.; Ju, S., *ACS Nano* **2014,** *8* (12), 12476-12482.

S2.	Yao, L.; Fang, X.; Gu, W.; Zhai, W.; Wan, Y.; Xie, X.; Xu, W.; Pi, X.; Ran, G.; Qin, G., *ACS Appl. Mater. Interfaces* **2017,** *9* (28), 24005-24010.

S3.	Jing, P.; Ji, W.; Zeng, Q.; Li, D.; Qu, S.; Wang, J.; Zhang, D., *Sci. Rep.* **2015,** *5* (1), 12499.

S4.	Kim, S.; Kim, J.; Kim, D.; Kim, B.; Chae, H.; Yi, H.; Hwang, B., *ACS Appl. Mater. Interfaces* **2019,** *11* (29), 26333-26338.

S5.	Zhang, N.; Qu, X.; Lyu, Q.; Wang, K.; Sun, X. W., *Photonics Res.* **2021,** *9* (10), 1979-1983.

S6.	Jiang, W.; Lee, S.; Zhao, K.; Lee, K.; Han, H.; Oh, J.; Lee, H.; Kim, H.; Koo, C. M.; Park, C., *ACS Nano* **2022,** *16* (6), 9203-9213.

S7.	Su, D.; Ding, T.; Gao, P.; Liu, H.; Song, Y.; Yuan, G.; He, X.; Meng, F.; Wang, S., *ACS Appl. Mater. Interfaces* **2024,** *16* (40), 54189-54198.
